# Preclinical Evaluation of [^155/161^Tb]Tb-Crown-TATE—A Novel SPECT Imaging Theranostic Agent Targeting Neuroendocrine Tumours

**DOI:** 10.3390/molecules28073155

**Published:** 2023-04-01

**Authors:** Luke Wharton, Scott W. McNeil, Helen Merkens, Zheliang Yuan, Michiel Van de Voorde, Gokce Engudar, Aidan Ingham, Helena Koniar, Cristina Rodríguez-Rodríguez, Valery Radchenko, Maarten Ooms, Peter Kunz, François Bénard, Paul Schaffer, Hua Yang

**Affiliations:** 1 Life Sciences Division, TRIUMF, 4004 Wesbrook Mall, Vancouver, BC V6T 2A3, Canada; lwharton@triumf.ca (L.W.);; 2 Department of Molecular Oncology, BC Cancer Research Institute, Vancouver, BC V5Z 1L3, Canada; 3 Nuclear Medicine Applications, Belgium Nuclear Research Center (SCK CEN), Boeretang, 200, 2400 Mol, Belgium; 4 Department of Physics and Astronomy, University of British Columbia, 6224 Agronomy Road, Vancouver, BC V6T 1Z1, Canada; 5 Faculty of Pharmaceutical Sciences, University of British Columbia, 2405 Wesbrook Mall, Vancouver, BC V6T 1Z3, Canada; 6 Department of Chemistry, University of British Columbia, 2036 Main Mall, Vancouver, BC V6T 1Z1, Canada; 7 Accelerator Division, TRIUMF, 4004 Wesbrook Mall, Vancouver, BC V6T 2A3, Canada; 8 Department of Chemistry, Simon Fraser University, Burnaby, BC V5A 1S6, Canada; 9 Department of Radiology, University of British Columbia, Vancouver, BC V5Z 1M9, Canada

**Keywords:** Terbium-155, Terbium-161, SPECT imaging, theranostics, NETs, PRRT

## Abstract

Terbium radioisotopes (^149^Tb, ^152^Tb, ^155^Tb, ^161^Tb) offer a unique class of radionuclides which encompass all four medicinally relevant nuclear decay modalities (α, *β*^+^, γ, *β*^−^/e^−^), and show high potential for the development of element-matched theranostic radiopharmaceuticals. The goal of this study was to design, synthesise, and evaluate the suitability of crown-TATE as a new peptide-conjugate for radiolabelling of [^155^Tb]Tb^3+^ and [^161^Tb]Tb^3+^, and to assess the imaging and pharmacokinetic properties of each radiotracer in tumour-bearing mice. [^155^Tb]Tb-crown-TATE and [^161^Tb]Tb-crown-TATE were prepared efficiently under mild conditions, and exhibited excellent stability in human serum (>99.5% RCP over 7 days). Longitudinal SPECT/CT images were acquired for ^155^Tb- and ^161^Tb- labelled crown-TATE in male NRG mice bearing AR42J tumours. The radiotracers, [^155^Tb]Tb-crown-TATE and [^161^Tb]Tb-crown-TATE, showed high tumour targeting (32.6 and 30.0 %ID/g, respectively) and minimal retention in non-target organs at 2.5 h post-administration. Biodistribution studies confirmed the SPECT/CT results, showing high tumour uptake (38.7 ± 8.0 %ID/g and 38.5 ± 3.5 %ID/g, respectively) and favourable tumour-to-background ratios. Blocking studies further confirmed SSTR2-specific tumour accumulation. Overall, these findings suggest that crown-TATE has great potential for element-matched molecular imaging and radionuclide therapy using ^155^Tb and ^161^Tb.

## 1. Introduction

Peptide receptor radionuclide therapy (PRRT) with complementary diagnostic imaging is a highly effective approach for the treatment of advanced neuroendocrine tumours (NETs) and has shown high response rates in patients previously refractory to conventional somatostatin therapy [1,2,3]. This approach involves the systemic administration of therapeutic radiopharmaceuticals which enable the selective localisation of ionising radiation to tumour sites through peptide-based molecular recognition of cell-surface receptors. The successful application of PRRT in the clinic is exemplified by [^177^Lu]Lu-DOTA-TATE (Lutathera^®^), which has shown high patient efficacy and was approved by the U.S. Food and Drug Administration (FDA) in 2018 following the NETTER-1 Phase III clinical trial. This treatment is suitable for early stage (G1-G2) progressive, well-differentiated gastroenteropancreatic (GEP) NETs [4]. A complete course of treatment (4 × 7.4 GBq, 8-week cycle) with [^177^Lu]Lu-DOTA-TATE achieves a median progression free survival of 28.4 months [4] and shows improvements in median overall survival (OS) by 11.7 months compared to the control group; however, this difference was not statistically significant. Additional treatment options are required for patients who experience relapse, show poor tolerance, or do not respond to treatment [5,6,7].

Targeted alpha therapy (TAT) utilising α-emitting radionuclides (^225^Ac, ^227^Th, ^230^U, ^213^Bi, ^211^At) is being intensively pursued in the emerging radiopharmaceutical sector as a potential alternative to conventional *β*-therapy for treatment of systemic malignancies, with several Phase II clinical trials underway ([^225^Ac]Ac-DOTA-TATE) [8,9]. The high potency of alpha radiation has achieved tumour regression in patients who did not respond to ^177^Lu treatments [10,11]. To fully capitalise on the potential benefits offered by TAT, accurate personalised dosimetry is crucial. Terbium radionuclides (^149^Tb, ^152^Tb, ^155^Tb, ^161^Tb) are particularly attractive in this context, owing to their diverse nuclear decay characteristics (α, PET, SPECT, *β*^−^/e^−^, respectively) and myriad of half-lives (4.12 h, 17.5 h, 5.32 d, 6.95 d, respectively), which may allow advancement of element-matched theranostic radiopharmaceuticals [10,11,12]. From this quartet of radioisotopes, ^155^Tb and ^161^Tb are a particularly appealing theranostic pair, with ^155^Tb (t_1/2_ = 5.32 d) being suitable for single photon emission computed tomography (SPECT) diagnostics and ^161^Tb (t_1/2_ = 6.95 d) being suitable for *β*-therapy. ^161^Tb shares similar decay characteristics to ^177^Lu and is being pursued as a more potent therapeutic radionuclide owing to the additional Meitner–Auger electron (MAE) emissions and low energy γ-ray emissions which are suitable for post-therapy whole-body SPECT scintigraphy [13,14]. Furthermore, ^155^Tb also generates several MAEs in its decay scheme which are of sufficient energy and intensity for therapeutic applications, making ^155^Tb a potential standalone theranostic radionuclide. In addition, expanding the repertoire of imaging radionuclides with longer half-lives (i.e., ^155^Tb (t_1/2_ = 5.32 d)) than the conventional clinically applied isotopes (i.e., ^18^F (t_1/2_ = 110 min), ^68^Ga (t_1/2_ = 68 min), ^99m^Tc (t_1/2_ = 6 h)) is another attractive feature of terbium radioisotopes and would enable diagnostic imaging studies at later timepoints which may be beneficial for determining the ultimate biological fate of long-lived therapeutic radiopharmaceuticals (^225^Ac, ^227^Th, ^230^U).

In a previous study, we introduced crown [15] as a new chelating ligand with impressive characteristics for coordinating large radiometal ions, specifically ^225^Ac. Crown was shown to form stable, kinetically inert metal complexes with [^225^Ac]Ac^3+^ under mild conditions (ambient temperature, 10 min); a clear advantage over the conventional gold-standard chelator DOTA which requires high temperatures and/or long reaction times to achieve quantitative radiolabelling [15]. Further studies of the corresponding bioconjugate, crown-αMSH, established the in vivo viability of the ^225^Ac-labelled chelator, demonstrating effective targeting of subcutaneous melanoma tumours and maintaining high in vivo stability in preclinical studies [15]. Ongoing studies involving an ^255^Ac-labelled bioconjugate for TAT of NETs are currently in progress [16]. 

To further explore the scope of this promising chelator, a new peptide-chelate bioconjugate targeting NETs, referred to as crown-TATE herein, was designed and synthesised. In this research, we investigate for the first time the suitability of crown-TATE for radiolabelling of both [^155^Tb]Tb^3+^ and [^161^Tb]Tb^3+^, and assess the kinetic inertness of the resulting complexes using human serum stability studies. Preclinical assessments of both [^155^Tb]Tb-crown-TATE and [^161^Tb]Tb-crown-TATE were undertaken using longitudinal quantitative SPECT/CT in tumour-bearing mice to establish the imaging potential for each of these radiotracers. Full biodistribution studies were performed on both blocked and unblocked groups to determine the in vivo performance of this new bioconjugate for targeting NETs. Altogether, this study demonstrates the capability of crown-TATE to chelate ^155^Tb and ^161^Tb effectively and stably, and its feasibility to selectively target AR42J NETs in mice. Overall, this research confirms the in vivo viability of these radiotracers as imaging agents for use in conjunction with ^225^Ac TAT or as standalone ^155^Tb/^161^Tb theranostics.

## 2. Results and Discussion

### 2.1. Synthesis of Crown-TATE

The majority of advanced, progressive NETs show overexpression of somatostatin receptors (SSTRs) on the cell surface and are therefore attractive targets for radionuclide imaging and therapy [1,2]. In humans, SSTRs are comprised of five subtypes (SSTR1-5), which are expressed in numerous organ systems including the gastrointestinal tract, pancreas, lungs, and renal organs. Several classes of SSTR2 targeting peptides have been developed including agonists (DOTA-TOC, DOTA-TATE) and antagonists (DOTA-LM3), both of which show high receptor binding affinity with low non-target organ toxicities [13,17,18,19]. The clinically applied bioconjugate DOTA-TATE (DOTA-[Tyr^3^]-octreotate) utilises a disulphide-bridged cyclic octapeptide which has been refined through numerous structure activity relationship studies for optimal binding towards SSTR2 [20,21,22]. This optimised peptide sequence was selected for conjugation to crown to enable direct comparison to a clinically established radiopharmaceutical.

**Figure 1:**
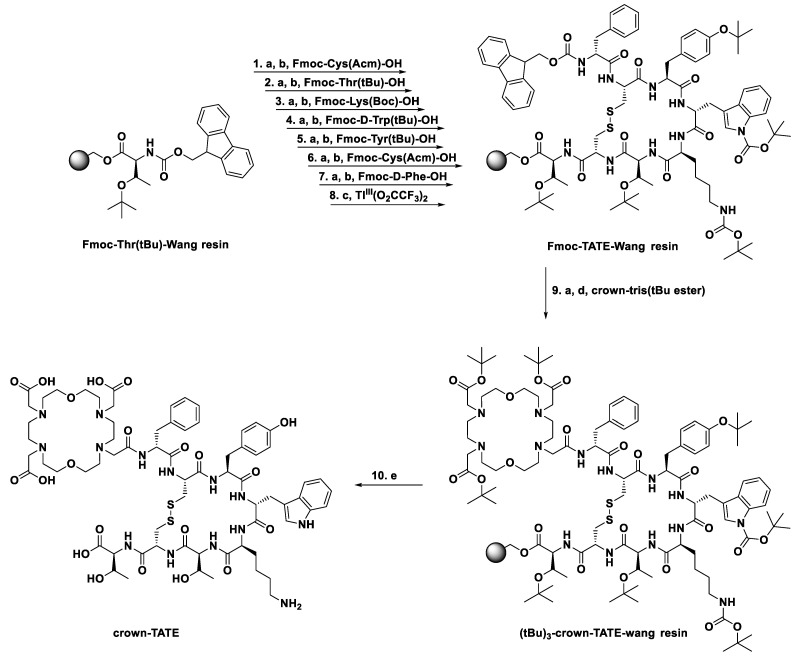
Solid phase peptide synthesis of crown-TATE. (a) Fmoc deprotection—20% piperidine in DMF; (b) amino acid coupling—PyBop (4 eq.), Oxyma Pure (4 eq.), DIPEA (15 eq.) in DMF, 2 h; (c) cyclisation—Tl(III)(O_2_CCF_3_)_2_ in DMF/anisole (19:1), 3 h; (d) chelator coupling—PyBop (4 eq.), Oxyma Pure (4 eq.), DIPEA (15 eq.) in DMF, 21 h; (e) deprotection and resin cleavage—TFA/TIPS/H_2_O/Thioanisole/Phenol (82.5:2.5:5:5:5), 3 h.

Crown-TATE was prepared using standardised Fmoc-based solid phase peptide synthesis (SPPS). Briefly, the linear peptide sequence—[DPhe-Cys(Acm)-Tyr(*t*Bu)-DTrp(*t*Bu)-Lys(Boc)-Thr(*t*Bu)-Cys(Acm)-Thr(*t*Bu)-OH]—was synthesised on Wang resin using PyBop/Oxyma Pure coupling methodology, Figure 1. The linear peptide was cyclised between residues Cys^2^ and Cys^7^ using thallium (III) trifluoroacetate, which simultaneously cleaves the S-acetamidomethyl (Acm) side chain protecting groups and generates the disulphide-bridged cyclic peptide [23,24]. Following Fmoc deprotection, the crown chelator (crown-tris (*t*Bu ester)) [15] was coupled to the free *N*-terminus using an extended reaction time (21 h) to ensure complete coupling. Global deprotection of the side chain protecting groups and cleavage from the resin was achieved using a standard cleavage cocktail to give the target bioconjugate which was isolated by reverse-phase high performance liquid chromatography (RP-HPLC). Crown-TATE was purified using RP-HPLC and the corresponding mass confirmed by high-resolution electrospray ionisation mass spectrometry (HR-ESI-MS). Characterisation data for crown-TATE is provided in the Supporting Information, Figure S1.

### 2.2. Radiochemistry

#### 2.2.1. Radionuclide Production

^155^Tb was produced via the proton-induced spallation of tantalum foil targets using the 480 MeV proton beamline at the Isotope Separator and Accelerator facility with Isotope Separation On-Line (ISOL) at TRIUMF (Canada) [25]. The ISAC/ISOL facility permits the production and separation of singly charged isobaric radioactive ion beams and allowed for the isolation of a single beam with mass-to-charge ratio of 155 A/q containing ^155^Tb (t_1/2_ = 5.32 d), ^155^Dy (t_1/2_ = 9.9 h), ^155^Ho (t_1/2_ = 48 min), and ^155^Er (t_1/2_ = 5.3 min). High purity ^155^Tb was obtained by beam implantation onto an aluminium target coated with an NH_4_Cl salt layer on the surface. After a 5-day cool-down period, which allowed for decay of ^155^Dy to ^155^Tb, and decay of short-lived ^155^Ho and ^155^Er, the activity was easily recovered by dissolution of the salt layer with de-ionised H_2_O to give [^155^Tb]Tb^3+^ in a form that was directly suitable for radiolabelling [25]. 

^161^Tb was produced via the neutron bombardment of enriched [^160^Gd][Gd_2_O_3_ (98.2%) targets using the high thermal neutron flux (3 × 10^14^ neutrons/cm^2^/s) BR2 reactor at SCK-CEN (Belgium) [26] and subsequently purified at TRIUMF using a semi-automated TRASIS system with solid phase extraction (SPE) resins (TK211, TK 212, TK221) [27]. 

#### 2.2.2. Radiolabelling Studies

**Figure 2:**
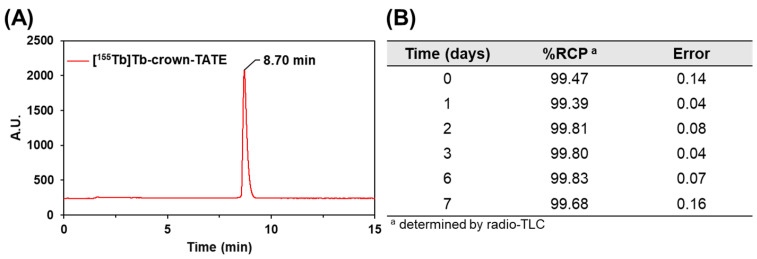
(**A**) Radio-HPLC trace of [^155^Tb]Tb-crown-TATE (19.4 MBq/nmol), prepared in NH_4_OAc buffer (0.5 M, pH 6.0) at ambient temperature after 10 min; >99% RCP. (**B**) Human serum stability challenge of [^161^Tb]Tb-crown-TATE (11.4 MBq/nmol), incubated at 37 °C, monitored over 7 days (*n* = 3); RCP determined by radio-TLC using SG-paper plates and EDTA (50 mM, pH 5.5) as eluent.

The radiolabelling properties of crown-TATE with [^155/161^Tb]Tb^3+^ were evaluated under mild conditions (ambient temperature, 10 min, pH 6.0) and showed quantitative radiolabelling with both [^155^Tb]Tb^3+^ and [^161^Tb]Tb^3+^, achieving molar activities of 19.4 MBq/nmol and 11.4 MBq/nmol, respectively. Quality control studies were undertaken using radio-HPLC which showed a single sharp peak for the [^155^Tb]Tb-crown-TATE product (T_R_ = 8.7 min) and no free [^155^Tb]Tb^3+^ activity (T_R_ = 1.0 min) in the final product (>99% radiochemical purity (RCP)), Figure 2A.

Human serum contains numerous endogenous proteins (e.g., albumin, transferrin) which can compete with the chelate for the binding of different metal ions. Prior to undertaking any preclinical studies, the kinetic inertness of [^161^Tb]Tb-crown-TATE was evaluated using a human serum stability challenge assay, Figure 2B. No evidence of transmetalation of bound activity to serum proteins was observed over the course of 7 days (>99.5% RCP); implying excellent overall stability.

### 2.3. Preclinical Studies 

#### 2.3.1. SPECT/CT Studies

**Figure 3:**
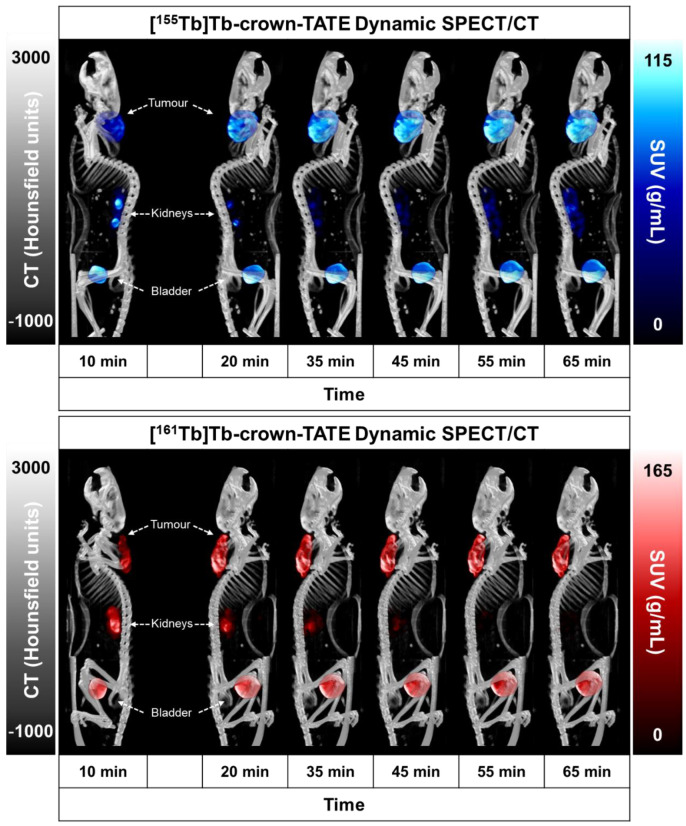
Maximum intensity projections (MIPs) from quantitative dynamic SPECT/CT scans recorded at 0–1 h post-administration of (**top**) [^155^Tb]Tb-crown-TATE (13.8 MBq, 0.71 nmol) and (**bottom**) [^161^Tb]Tb-crown-TATE (7.95 MBq, 0.70 nmol) in male NRG mice bearing AR42J tumour xenografts (left-shoulder).

**Figure 4:**
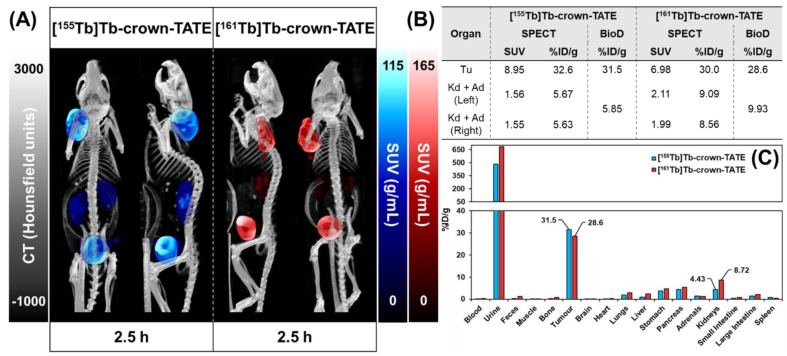
Summary of preclinical data for imaging mice bearing AR42J tumour xenografts at 2.5 h post-administration of [^155^Tb]Tb-crown-TATE (13.8 MBq, 0.71 nmol) and [^161^Tb]Tb-crown-TATE (7.95 MBq, 0.70 nmol). (**A**) Viewpoints of MIPs from quantitative static SPECT/CT. (**B**) Comparison of the activity-concentrations in ROIs measured via quantitative SPECT/CT vs. ex vivo biodistribution studies; Tu: Tumour, Kd: Kidneys, Ad: Adrenal glands. (**C**) Full biodistribution results for imaging mice at 2.5 h p.i. Image studies were performed using two replicates (*n* = 2) per radiotracer.

Preclinical SPECT/CT studies were undertaken to evaluate the potential of [^155^Tb]Tb-crown-TATE and [^161^Tb]Tb-crown-TATE for imaging pancreatic exocrine tumours. The ^155^Tb/^161^Tb-labelled radiotracers were each administered to male NRG mice bearing AR42J tumour xenografts (left-shoulder) and a series of dynamic quantitative SPECT/CT scans were acquired from 0 to 2.5 h post-administration, Figure 3 and Figure 4. From the quantitative SPECT/CT scans, mean standardised uptake values (SUV_mean_) were extracted for regions of interest (ROIs) in tumour, kidneys, and bladder. The quantitative imaging studies enabled direct measurement of the activity concentrations (%ID/g) in ROIs in the SPECT/CT scans over time which can be compared to the ex vivo biodistribution results obtained after euthanising the mice following acquisition of the final SPECT/CT scan (2.5 h p.i.). Full biodistribution results for the imaging studies are provided in the Supplementary Materials, Figure S3 and Table S1.

The dynamic SPECT/CT studies showed rapid uptake of [^155^Tb]Tb-crown-TATE in AR42J tumours (left-shoulder) beginning at 10 min post-administration (SUV_mean_ = 5.19 g/mL) which increases over time reaching a maximum at 2.5 h p.i. (SUV_mean_ = 8.95 g/mL), Table 1. Quantification of the SPECT/CT scans revealed a tumour uptake of 32.6 %ID/g at 2.5 h p.i., which was consistent with the ex vivo biodistribution measurements (31.5 %ID/g). As anticipated, the SPECT/CT imaging results for [^161^Tb]Tb-crown-TATE were highly comparable to those of the ^155^Tb-labelled radiotracer, exhibiting a similar pharmacokinetic profile and high tumour uptake (30.0 %ID/g at 2.5 h p.i.), which was further confirmed in the biodistribution studies (28.6 %ID/g).

**Figure 5:**
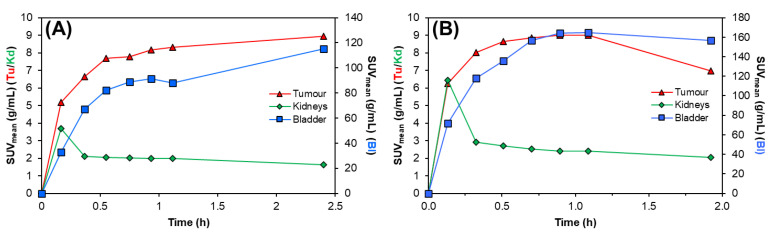
Representative time–activity curves for (**A**) [^155^Tb]Tb-crown-TATE (13.8 MBq, 0.71 nmol) and (**B**) [^161^Tb]Tb-crown-TATE (7.95 MBq, 0.70 nmol) in male NRG mice bearing AR42J tumour xenografts. Tu: Tumour; Kd: Kidneys; Ad: Adrenal glands; Bl: Bladder.

Both radiotracers showed fast clearance from the bloodstream, with rapid elimination through the renal pathway, as evidenced by activity in the kidneys and bladder, giving high contrast images. This is further supported by the time–activity curves, Figure 5, where most of each radiotracer was cleared from circulation and tumour uptake reached a plateau within 0.5 h p.i., as shown in Table 1. Comparatively, the preclinical SPECT studies with ^155^Tb yielded superior imaging quality over ^161^Tb, with higher image contrast and lower signal noise. However, these studies clearly show the suitability of ^161^Tb for performing dosimetry and patient monitoring studies following administration of ^161^Tb therapy treatments, as is standard practice for ^177^Lu therapies. 

#### 2.3.2. Biodistribution Studies

**Figure 6:**
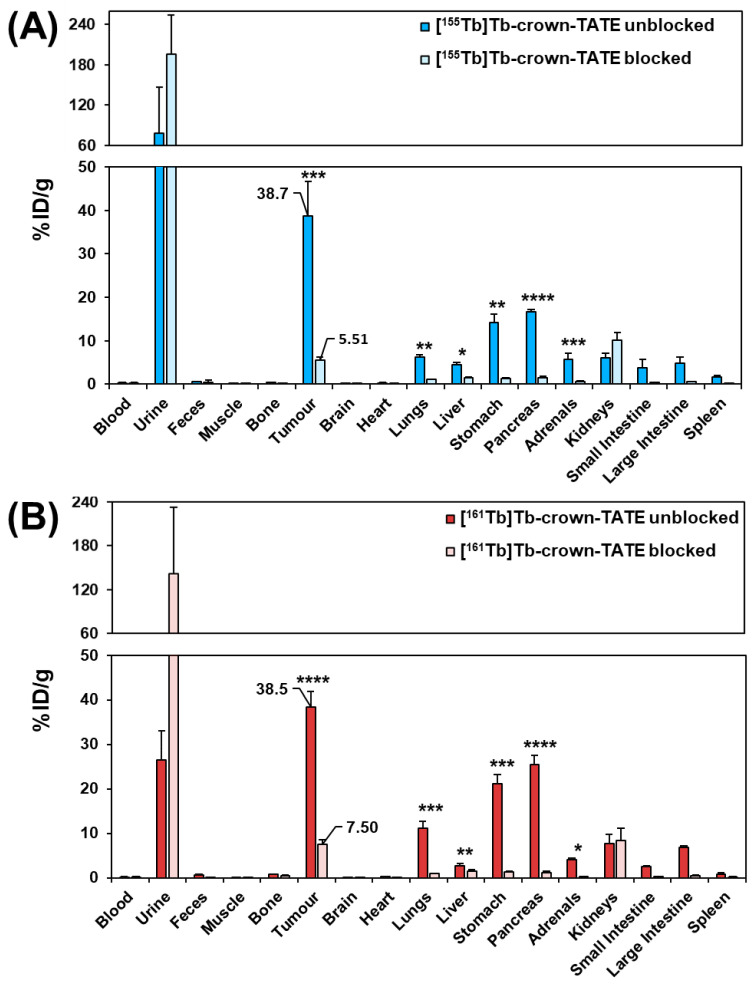
Biodistribution results in male NRG mice bearing AR42J tumours at 2 h post-administration of the following: (**A**) [^155^Tb]Tb-crown-TATE (~175 kBq, 93 pmol/animal) (*n* = 4) (unblocked) and [^155^Tb]Tb-crown-TATE (~175 kBq, 93 pmol/animal) (blocked with DOTATOC (23 nmol/animal)) (*n* = 3); (**B**) [^161^Tb]Tb-crown-TATE (~850 kBq, 76 pmol/animal) (unblocked) (*n* = 4) and [^161^Tb]Tb-crown-TATE (~850 kBq, 76 pmol) (blocked with DOTATOC (23 nmol/animal) (*n* = 3). (* = *p* < 0.05, ** = *p* < 0.01, *** = *p* < 0.001, **** = *p* < 0.0001).

Full biodistribution studies were performed for the ^155^Tb- and ^161^Tb- radiolabelled bioconjugates in tumour-bearing mice at 2 h post-administration, Figure 6. Mice were administered with either [^155^Tb]Tb-crown-TATE (~175 kBq, 93 pmol per animal) or [^161^Tb]Tb-crown-TATE (~850 kBq, 76 pmol per animal). Additional blocking control studies were undertaken to confirm SSTR2 specific uptake by co-administration of excess unlabelled DOTA-TOC (23 nmol per animal) with each radiotracer. Full biodistribution results for each radiotracer are provided in the Supporting Information, Figure S4 and Table S2.

The ex vivo biodistribution results show good consistency with the SPECT/CT studies, with high tumour uptake for both [^155^Tb]Tb-crown-TATE (38.7 ± 8.0 %ID/g) and [^161^Tb]Tb-crown-TATE (38.5 ± 3.5 %ID/g), and elimination primarily through the renal pathway. Blocking control studies confirmed tumour uptake of each radiotracer was SSTR2 specific (*p* < 0.001 and *p* < 0.0001, respectively). Additionally, statistically significant decreases in radiotracer uptake by non-target organs expressing SSTR2 (lungs, stomach, pancreas, adrenals glands, and intestines) are observed in the blocking studies, further confirming SSTR2 specific uptake. Although [^155^Tb]Tb-crown-TATE showed an 86% reduction in tumour uptake with co-administration of the blocking agent, and a comparable decrease of 81% was demonstrated for [^161^Tb]Tb-crown-TATE, the blocking groups did not demonstrate complete knock-down of tumour uptake for [^155^Tb]Tb-crown-TATE (5.51 %ID/g) or [^161^Tb]Tb-crown-TATE (7.50 %ID/g); this result was anticipated since the blocking agent was co-administered with each radiotracer injection, rather than using a pre-blocking approach. This is comparable to previous reports which demonstrated that co-injection of unlabelled TATE decreased tumour uptake of [^64^Cu]Cu-DOTATATE by 83% in mice at 4 h post-injection [28].

Low kidney uptake was observed for the ^155^Tb- and ^161^Tb-labelled tracers (6.13 ± 0.99 %ID/g and 7.71 ± 2.11 %ID/g, respectively; *p* = 0.329). Favourable tumour-to-kidney ratios were achieved for both radiotracers (6.3:1 for [^155^Tb]Tb-crown-TATE and 5.0:1 for [^161^Tb]Tb-crown-TATE). Bone uptake for [^155^Tb]Tb-crown-TATE and [^161^Tb]Tb-crown-TATE was low (0.34 ± 0.06 %ID/g and 0.73 ± 0.09 %ID/g, respectively) implying good radiotracer stability at this timepoint. The radiotracers show the typical pharmacokinetic profile exhibited by TATE-based bioconjugates, with moderate uptake in organs expressing SSTR2 (kidneys, lungs, stomach, pancreas, intestines, and adrenal glands), which is comparable to the biodistribution of [^177^Lu]Lu-DOTA-TATE [29].

## 3. Materials & Methods

### 3.1. General

All solvents and reagents were purchased from commercial suppliers (Sigma-Aldrich (Markham, ON, Canada), (Alfa Aesar, Tewksbury, MA, USA) and used directly without further purification. Crown-tris(*t*Bu ester) was prepared as previously described [15]. Radiolabelling studies were monitored using instant thin-layer chromatography (iTLC) with silica gel (SG)-impregnated paper TLC plates (Agilent technologies, Santa Clara, CA, USA). TLC imaging was performed using an AR-2000 imaging scanner (Eckert & Ziegler, Berlin, Germany) equipped with P-10 gas, and radiochemical yields (RCYs) analysed using WinScan V3_14 software. Radio-HPLC was carried out using an Agilent 1200 instrument equipped with a Phenomenex Luna C18 reverse phase column (100 × 46 mm, 5 μm) and a GABI star radioactive HPLC flow monitor (Elysia-raytest GmbH, Germany). Radioactivity was quantified using a calibrated high-purity germanium (HPGe) detector (Mirion Technologies (Canberra) Inc., Meriden, CT, USA) with Genie 2000 software. All work with radionuclides at TRIUMF was undertaken in shielded fume hoods to minimise the dose to experimenters (and special precautions were used to prevent contamination) under nuclear energy worker (NEW) status earned by attending TRIUMF’s Advanced Radiation Protection course and passing the final exam. Peptides were prepared using a Focus Xi semi-automated solid phase peptide synthesiser (AAPPTec, Louisville, KY, USA). SPECT/CT studies were performed using a multimodal VECTor/CT system (MILabs, Houten, Netherlands) in combination with an extra ultra-high sensitivity (XUHS) pinhole collimator (2-mm). Image analysis was performed using AMIDE (v. 1.0.5) software [30].

### 3.2. Synthesis of Crown-TATE

Crown-TATE was prepared using standardised Fmoc-based solid phase peptide synthesis. The linear peptide sequence—[DPhe-Cys(Acm)-Tyr(*t*Bu)-DTrp(*t*Bu)-Lys(Boc)-Thr(*t*Bu)-Cys(Acm)-Thr(*t*Bu)-OH]—was generated using PyBop/Oxyma Pure peptide coupling methodology on Wang resin preloaded with Fmoc-Thr(*t*Bu)-OH. Prior to coupling the first amino acid, the preloaded Wang resin was swelled in DMF (5 mL), CH_2_Cl_2_ (5 mL), and then DMF (5 mL) again. Fmoc-deprotection of the *N*-terminus was achieved using 20% piperidine in DMF (2 × 10 mL) and confirmed by positive Kaiser test. Successive amino acids were coupled to the *N*-terminus using a mixture consisting of the amino acid (4 eq.), PyBop (4 eq.), Oxyma Pure (4 eq.), and DIPEA (15 eq.) dissolved in DMF (5 mL). Each amino acid was preactivated for 5 min at RT before addition to the peptidyl resin. Coupling reactions were mechanically shaken for 2–3 h at RT under a N_2_ atmosphere, and reaction completion was confirmed using a negative Kaiser test. The linear sequence was cyclised between Cys^2^ and Cys^7^ on-resin by addition of thallium(III) trifluoroacetate (1.2 eq.) in DMF/anisole (19:1) to the peptidyl resin at 0 °C and shaking for 2 h. Coupling of the crown chelator was performed on-resin. Crown-tris(*t*Bu ester) (4 eq.), PyBop (4 eq.), Oxyma Pure (4 eq.), and DIPEA (15 eq.) were dissolved in DMF (5 mL), and shaken for 5 min at RT. The solution was transferred immediately to the reaction vessel, purged with N_2_ gas, and the resulting mixture was shaken at RT for 21 h. Global deprotection and resin cleavage was achieved using a cleavage cocktail consisting of TFA/TIPS/H_2_O/thioanisole/phenol (82.5:2.5:5:5:5). After 3 h at RT, the resin was filtered, and the peptide precipitated using diethyl ether. The crude white precipitate was separated by centrifuge, washed with cold diethyl ether (3 × 10 mL), and re-dissolved in H_2_O (2 mL). The crude peptide solution was lyophilised and redissolved in H_2_O (0.1% TFA) prior to HPLC purification. Crown-TATE was purified via RP-HPLC using isocratic conditions (23% MeCN (0.1% TFA) in deionised H_2_O (0.1% TFA); 30 min.; T_R_ = 7.1 min). HR-ESI-MS (calculated for [C_69_H_98_N_14_O_21_S_2_+H]^+^: 1523.6545, [C_69_H_98_N_14_O_21_S_2_+2H]^2+^: 762.3309, [C_69_H_98_N_14_O_21_S_2_+H+K]^2+^: 781.3088) found at [C_69_H_98_N_14_O_21_S_2_+2H]^2+^: 762.3385, [C_69_H_98_N_14_O_21_S_2_+H+K]^2+^: 781.3123.

### 3.3. Radionuclide Production

#### 3.3.1. [^155^Tb]Tb^3+^

^155^Tb was produced via proton-induced spallation of tantalum foil targets using the 480 MeV proton beamline at the Isotope Separator and Accelerator facility with Isotope Separation On-Line (ISOL) at TRIUMF (Canada) [25]. Radioisotopes produced via the ISAC/ISOL technique are liberated from the target material by diffusion and effusion processes under ultra-high vacuum at high temperature (2300 °C) [31]. Released radioisotopes are then ionised in a combined surface and laser ion source and accelerated under high voltage to produce a heterogenous radioactive ion beam which is passed through a pre-separator magnet, followed by a high-resolution mass separator magnet to give a singly charged, isobaric ion beam. For ^155^Tb, a radioactive ion beam with mass-to-charge ratio of 155 A/q is separated. The ion beam was implanted onto aluminium targets with an NH_4_Cl salt layer deposited on the surface and retrieved following a 5-day cool-down period after implantation which allows for decay of short-lived radionuclides (^155^Ho, ^155^Er) and ingrowth of ^155^Tb from ^155^Dy decay. The activity is then easily recovered by dissolution of the NH_4_Cl salt layer using de-ionised H_2_O and yields high-purity [^155^Tb]Tb^3+^ at high specific activity (~ 175 MBq/184 μL) which can be used directly without additional purification [25,32]. Typical production yields of ^155^Tb were ~ 300 MBq, with no detectable isotopic impurities as determined by gamma spectroscopy.

#### 3.3.2. [^161^Tb]Tb^3+^

^161^Tb was produced via the neutron bombardment of enriched [^160^Gd][Gd_2_O_3_ (98.2%) targets using the high thermal neutron flux (3 × 10^14^ neutrons/cm^2^/s) BR2 reactor at SCK-CEN (Belgium) [26]. After cooling, the ^161^Tb activity was purified using a semi-automated TRASIS system equipped with TrisKem solid phase extraction (SPE) resins (TK211, TK212, TK221 (Triskem, Bruz, France) at TRIUMF (Canada) as described by McNeil et al. [27]. A radionuclidic purity of > 99% was determined using ICPMS for ^161^Tb purified using this approach.

### 3.4. Radiolabelling Studies

Initial radiolabelling studies of crown-TATE with [^155^Tb]Tb^3+^ and [^161^Tb]Tb^3+^ were performed to establish suitable labelling conditions and quality control methods. [^155^Tb]Tb^3+^ (40–70 kBq, 2 μL) or [^161^Tb]Tb^3+^ (70–100 kBq, 2 μL) were added to solutions of crown-TATE (10^−4^ M, 10 μL) in NH_4_OAc buffer (0.5 M, pH 6.0, 88 μL); V_T_ = 100 μL. The final reaction pH (~6.0) was confirmed by spotting small aliquots of the reaction solution (~2–3 μL) on pH test strips. Reactions were allowed to stand at ambient temperature for 10 min, and then monitored by radio-TLC using SG-paper iTLC plates and EDTA (50 mM, pH 5.5) as mobile phase. Under these conditions free [^155/161^Tb]Tb^3+^ is bound by EDTA and migrates with the solvent front (R*_f_* = 0.9–1.0), while [^155/161^Tb]Tb-crown-TATE remains at the baseline (R*_f_* = 0.0–0.1). For all radiolabelling experiments, a separate control reaction was performed to assess the formation of any metal-hydroxo species or colloidal particles under the conditions tested. [^155^Tb]Tb^3+^ (40–70 kBq, 2 μL) or [^161^Tb]Tb^3+^ (70–100 kBq, 2 μL) were added to solutions of NH_4_OAc buffer (0.5 M, pH 6.0, 98 μL) without any chelator present. The reaction solutions were treated under the same conditions for each radiolabelling experiment and monitored using radio-TLC under the same conditions outlined for [^155/161^Tb]Tb-crown-TATE. Under these conditions, colloidal terbium or metal-hydroxo species would remain bound to the baseline of the SG-paper iTLC plates (R*_f_* = 0.0), while free [^155/161^Tb]Tb^3+^ migrates with the solvent front (R*_f_* = 0.9–1.0). In all radiolabelling studies of crown-TATE, the control reactions showed no evidence of colloidal terbium or metal-hydroxo species when performing these studies in NH_4_OAc buffer below pH 6.5. Representative radio-TLC traces for [^155^Tb][Tb-crown-TATE], [^161^Tb]Tb-crown-TATE], and control reactions are provided in the Supporting Information, Figure S2.

### 3.5. Radio-HPLC

Quality control studies for the ^155^Tb- and ^161^Tb- labelled radiotracers were performed by analytical radio-HPLC, using a Phenomenex Luna C18 reverse phase column (100 × 4.6 mm, 5 µm) and A: 0.1% TFA in water, B: 0.1% TFA in acetonitrile; gradient: 100% A to 100% B over 15 min, flow rate: 1 mL/min, T_R_ = 8.7 min.

### 3.6. Human Serum Stability

For human serum stability challenge studies, a high activity stock solution of [^161^Tb]Tb-crown-TATE (~1 MBq, 100 μL) was prepared using a similar procedure as outlined above. Aliquots of [^161^Tb]Tb-crown-TATE (~300 kBq, 30 μL) were diluted into pooled human serum (270 μL) and incubated at 37 °C for 7 days with gentle mechanical shaking. All studies were performed in triplicate (*n* = 3). The radiochemical purity was determined using radio-TLC measurements under the same conditions as outlined above.

### 3.7. Preclinical Studies

#### 3.7.1. Tumour Inoculation

Male NRG mice (24-weeks old) were inoculated with AR42J exocrine pancreatic tumour cells (3 × 10^6^) on the left shoulder and waited for tumours to grow to reach 8–10 mm in diameter for subsequent experiments. 

#### 3.7.2. Radiotracer Preparation

All sample preparations were performed using low retention pipette tips and low-protein binding Eppendorf tubes to minimise losses of the radiolabelled bioconjugates through surface adsorption. [^155^Tb]Tb-crown-TATE was prepared by addition of [^155^Tb]Tb^3+^ (19.5 MBq, 35 μL) to a solution containing NH_4_OAc (0.5 M, pH 6.0, 10 μL) and ultra-pure deionised H_2_O (10 μL). Crown-TATE (10^−3^ M, 1 μL, 1 nmol) was added and the reaction mixture agitated at 40 °C for 30 min to ensure quantitative radiolabelling. Reaction completion was confirmed using radio-TLC. To minimise the presence of any free metal ions and other impurities in the final preparation, the entire solution of quantitatively radiolabelled [^155^Tb]Tb-crown-TATE (60 μL) was loaded onto a Sep-Pak^®^ C18 Plus Light Cartridge (Waters^TM^) (pre-conditioned with EtOH (5 × 1 mL), then saline (5 × 1 mL)). The reaction vessel was rinsed with saline (2 × 200 μL) and loaded onto the Sep-Pak cartridge to ensure quantitative transfer. The Sep-Pak^®^ cartridge was washed with saline (5 mL) and the purified product eluted with an EtOH/saline solution (9:1; 2 × 500 μL). Purified [^155^Tb]Tb-crown-TATE (15.3 MBq) fractions were concentrated to ~50 μL under N_2_ gas stream and diluted with injectable saline to give suitable doses for preclinical studies. Quality control studies on the final preparation was performed using radio-TLC, radio-HPLC, and gamma spectroscopy. A similar protocol was applied for the preparation of [^161^Tb]Tb-crown-TATE. Final solution after purification: [^161^Tb]Tb-crown-TATE (14.9 MBq). 

#### 3.7.3. SPECT/CT Studies

To evaluate the suitability of [^155^Tb]Tb-crown-TATE and [^161^Tb]Tb-crown-TATE for imaging NETs, preclinical studies were conducted with male NRG mice bearing AR42J pancreatic exocrine tumour xenografts. For imaging studies experiments were performed using two replicates (*n* = 2) per radiotracer. Mice were immobilised using a Tailveiner restrainer (Braintree Scientific Inc., Braintree, MA, USA), and administered with [^155^Tb]Tb-crown-TATE (13.8 MBq, 0.71 nmol, 100 μL) or [^161^Tb]Tb-crown-TATE (7.98 MBq, 0.70 nmol, 100 μL) via intravenous injection to the lateral tail vein. Mice were anaesthetised and maintained under a continuous stream of isoflurane (1.5–2.0% in oxygen) throughout the acquisition of the SPECT/CT scans, and their animal body temperature was kept using a heated animal bed. Quantitative dynamic SPECT/CT scans were acquired over the first 1 h post-injection (p.i.) of [^155^Tb]Tb-crown-TATE or [^161^Tb]Tb-crown-TATE using a VECTor/CT multimodal preclinical scanner (MILabs, Netherlands) with an extra ultra-high sensitivity (XUHS) pinhole collimator (2 mm) [33,34]. After the first scan, mice were allowed to roam freely in their cages, and further static SPECT/CT scans were acquired at 2.5 h p.i. SPECT data were reconstructed with the pixel-based ordered subset expectation maximisation (POSEM) algorithm, with a voxel size of 0.4 mm^3^, 16 subsets, and 6 iterations. For ^155^Tb, three photopeaks were detected: 44, 85, and 106 keV, while for ^161^Tb, the photopeaks were 25, 47, and 75 keV. Reconstructions were carried out using photopeaks of 44 and 47 keV, respectively, for ^155^Tb and ^161^Tb, with a spectral width of 50%. Each imaging scan was decay corrected to the time of injection and recorded counts were adjusted using attenuation factors determined from CT scans at each time point. After acquisition of the final SPECT/CT scans, mice were kept under isoflurane and promptly euthanised by CO_2_ asphyxiation, followed by cardiac puncture to recover blood activity, and relevant organs were harvested for biodistribution studies. Point source measurements of known samples of ^155^Tb and ^161^Tb were used to determine calibration factors relating counts/voxel to activity concentration prior to performing the preclinical studies. From the quantitative SPECT/CT scans, mean standardised uptake values (SUV_mean_) were extracted for volumes of interest (VOIs) in the tumour, kidneys, and bladder, using AMIDE software (v. 1.0.5) [30]. SUV_mean_ is defined as follows: SUV_mean_ (g/mL) = [radioactivity concentration (MBq/mL)]/[injected dose (MBq)/animal weight (g)]. The %ID/g (injected dose/gram body tissue) in relevant organs was calculated by assuming a tissue density of 1 g/mL directly from the quantitative SPECT/CT scans using the following formula: %ID/g = [radioactivity concentration (MBq/mL) in VOIs]/[injected dose (MBq)] × 100. Maximum intensity projections (MIPs) for each time-point were generated using AMIDE software [30], where a Gaussian filtering of FWHM = 2 mm was used for image rendering.

#### 3.7.4. Biodistribution Studies

For biodistribution studies, experiments were performed using four replicates (*n* = 4) for each unblocked group and three replicates (*n* = 3) in the blocked groups. [^155^Tb]Tb-crown-TATE (~175 kBq, 93 pmol, 100 μL) or [^161^Tb]Tb-crown-TATE (~850 kBq, 76 pmol, 100 μL) was administered intravenously via the lateral tail using a Tailveiner restrainer. After administration, mice were allowed to roam freely in their cages. Biodistribution studies were performed at 2 h post-administration of [^155^Tb]Tb-crown-TATE or [^161^Tb]Tb-crown-TATE. Mice were euthanised by CO_2_ asphyxiation under 2% isoflurane anaesthesia, followed by cardiac puncture to recover activity in the blood pool. Organs of interest were completely harvested, weighed, and the activity measured using a gamma counter (Packard Cobra II, Perkin Elmer, Waltham, MA, USA). To account for organs that could not be fully extracted (such as blood, muscle, and bone), standardised organ weights for age- and sex-matched mice were used to correct the data [35,36]. The results are reported as the percentage of the injected dose per gram (%ID/g) and per organ (%ID/organ).

### 3.8. Statistical Analysis 

Statistical analysis was performed in Microsoft Excel and presented as average ± standard deviation. *p*-values were calculated using Welch’s *t*-test for two-samples with unequal variances.

## 4. Conclusions

While it may seem reasonable to expect that the biodistribution and pharmacokinetic profiles of the same bioconjugate labelled with either [^155^Tb]Tb^3+^ or [^161^Tb]Tb^3+^ would be identical, it is important to validate this assumption, since the production methods used for each radionuclide involve different nuclear reactions (proton spallation vs. neutron irradiation) and purification strategies (ISOL vs. SPE/HPIC), which result in products of differing radionuclidic purity and specific activities. 

In this proof-of-principle study, crown-TATE was synthesised for the first time and evaluated as a new bioconjugate targeting SSTR2-positive NETs. The results showed that crown-TATE can be efficiently radiolabelled with both [^155^Tb]Tb^3+^ and [^161^Tb]Tb^3+^ under mild conditions, and in vivo assessment demonstrated excellent tumour imaging contrast with minimal background organ uptake. Our investigation demonstrates the capability of crown-TATE to provide two new element-matched radiotracers which exhibit equivalent biodistribution and pharmacokinetic properties in vivo when radiolabelled with either ^155^Tb or ^161^Tb. The interchangeable metal complexation properties of the crown chelator provide a suitable platform for developing ^155^Tb as a diagnostic companion for ^225^Ac targeted alpha therapy. In addition, the favourable imaging properties of both terbium radionuclides will enable accurate staging of disease progression and assessment of patient suitability for treatment using ^155^Tb, as well as post-therapy scintigraphy following ^161^Tb administration. Further work focused on ^225^Ac-labelled crown-TATE is of high interest and will expand the applicability of this bioconjugate for more effective and personalised treatments of NETs. Additional therapy studies involving [^161^Tb]Tb-crown-TATE are the subject of ongoing research, which will allow direct comparison of the therapeutic efficacy of Meitner–Auger electron emitters versus alpha emitters.

## Figures and Tables

**Table 1:** Comparison of the activity-concentrations in ROIs as a function of time measured via quantitative SPECT/CT vs. ex vivo biodistribution studies for imaging mice administered with [^155^Tb]Tb-crown-TATE (13.8 MBq, 0.71 nmol) or [^161^Tb]Tb-crown-TATE (7.95 MBq, 0.70 nmol).

Time (h)	Tumour			Kidneys + Adrenals		
	SPECT		BioD	SPECT		BioD
	SUV_mean_	%ID/g	%ID/g	SUV_mean_	%ID/g	%ID/g
[^155^Tb]Tb-crown-TATE						
0	0	0	-	0	0	-
0.17	5.19	18.9	-	3.69	13.4	-
0.37	6.63	24.1	-	2.1	7.64	-
0.55	7.68	27.9	-	2.05	7.45	-
0.75	7.77	28.3	-	2.02	7.34	-
0.93	8.15	29.6	-	1.98	7.2	-
1.12	8.32	30.3	-	1.98	7.19	-
2.5	8.95	32.6	31.6	1.64	5.96	5.85
[^161^Tb]Tb-crown-TATE						
0	0	0	-	0	0	-
0.13	6.27	27.0	-	6.44	27.8	-
0.32	8.02	34.6	-	2.93	12.6	-
0.51	8.63	37.2	-	2.71	11.7	-
0.70	8.86	38.2	-	2.53	10.9	-
0.90	9.01	38.8	-	2.42	10.4	-
1.09	9.00	38.8	-	2.40	10.4	-
1.92	6.98	30.1	28.6	2.05	8.82	9.93

## Data Availability

Data sets analysed and reported here are available upon requested from the corresponding author.

## Supplementary Materials

The following supporting information can be downloaded at https://www.mdpi.com/article/10.3390/molecules28073155/s1; supplementary information for the radiolabelling experiments and preclinical SPECT/CT and biodistribution studies is provided here.

## Sample Availability

Samples of the compound crown-TATE is available from the authors.

## Abbreviations

%ID/g	% injected dose per gram tissue
CT	Computed tomography
GEP	Gastroenteropancreatic
HPIC	High pressure ion chromatography
HPLC	High performance liquid chromatography
ISAC	Isotope separator and accelerator
ISOL	Isotope separation on-line
MAE	Meitner–Auger electron
NETs	Neuroendocrine tumours
OS	Overall survival
PET	Positron emission tomography
PFS	Progression free survival
RCP	Radiochemical purity
RCY	Radiochemical yield
PRRT	Peptide receptor radionuclide therapy
ROIs	Regions of interest
SPE	Solid-phase extraction
SPECT	Single photon emission computed tomography
SSTR2	Somatostatin receptor-2
SUV	Standardised uptake value
TAT	Targeted alpha therapy
UHS	Ultra-high sensitivity
VECTor	Versatile emission computed tomography
