# Supplementary material for: Preclinical Evaluation of [155/161Tb]Tb-Crown-TATE—A Novel SPECT Imaging Theranostic Agent Targeting Neuroendocrine Tumours
Source: Molecules. 2023 Apr 1;28(7):3155. doi: 10.3390/molecules28073155 (PMC10095901; doi:10.3390/molecules28073155)
Supplement: Supplementary file 1 [file molecules-28-03155-s001.zip › molecules-2280861-supplementary.pdf]

Supporting Information for:

Preclinical Evaluation of [$^{155/161}$Tb]Tb-Crown-TATE—A Novel SPECT Imaging Theranostic Agent Targeting Neuroendocrine Tumours

Luke Wharton [1], Scott W. McNeil [1], Helen Merkens [2], Zheliang Yuan [1], Michiel Van de Voorde [3], Gokce Engudar [1], Aidan Ingham [1], Helena Koniar [1,4], Cristina Rodríguez-Rodríguez [4,5], Valery Radchenko [1,6], Maarten Ooms [3], Peter Kunz [7,8], François Bénard [2,9], Paul Schaffer [1,8,9] and Hua Yang [1,8,*]

1 Life Sciences Division, TRIUMF, 4004 Wesbrook Mall, Vancouver, BC V6T 2A3, Canada; lwharton@triumf.ca (L.W.)
2 Department of Molecular Oncology, BC Cancer Research Institute, Vancouver, BC V5Z 1L3, Canada;
3 Nuclear Medicine Applications, Belgium Nuclear Research Center (SCK CEN), Boeretang, 200, 2400 Mol, Belgium
4 Department of Physics and Astronomy, University of British Columbia, 6224 Agronomy Road, Vancouver, BC V6T 1Z1, Canada
5 Faculty of Pharmaceutical Sciences, University of British Columbia, 2405 Wesbrook Mall, Vancouver, BC V6T 1Z3, Canada
6 Department of Chemistry, University of British Columbia, 2036 Main Mall, Vancouver, BC V6T 1Z1, Canada
7 Accelerator Division, TRIUMF, 4004 Wesbrook Mall, Vancouver, BC V6T 2A3, Canada
8 Department of Chemistry, Simon Fraser University, Burnaby, BC V5A 1S6, Canada
9 Department of Radiology, University of British Columbia, Vancouver, BC V5Z 1M9, Canada
* Correspondence: hyang@triumf.ca

Table of Contents

Synthesis and Characterisation 3

Radiochemistry Studies 4

Radio-TLCs 4

Preclinical Studies 5

SPECT Biodistribution Studies 5

Full Biodistribution Studies 6

Synthesis and Characterisation

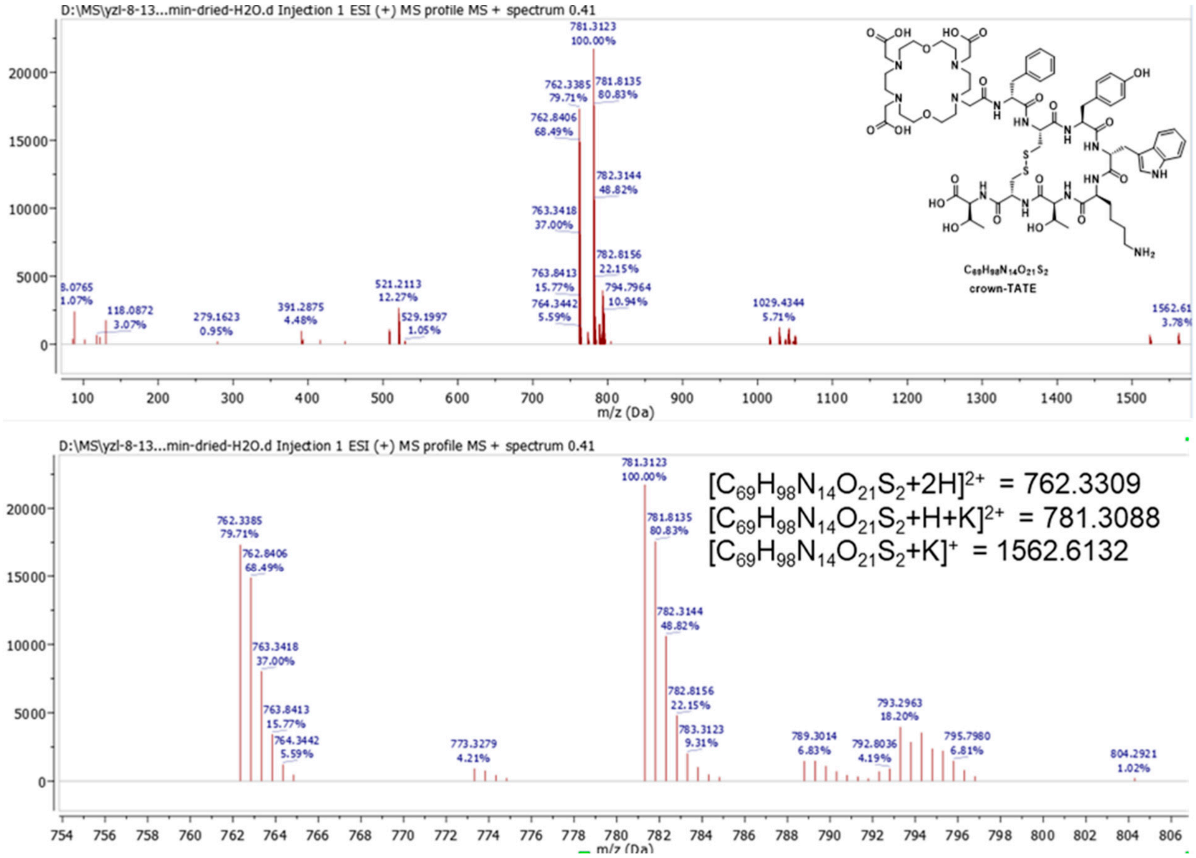

Figure S1. High-resolution electrospray ionisation mass spectrometry characterisation of crown-TATE.

Radiochemistry Studies

Radio-TLCs

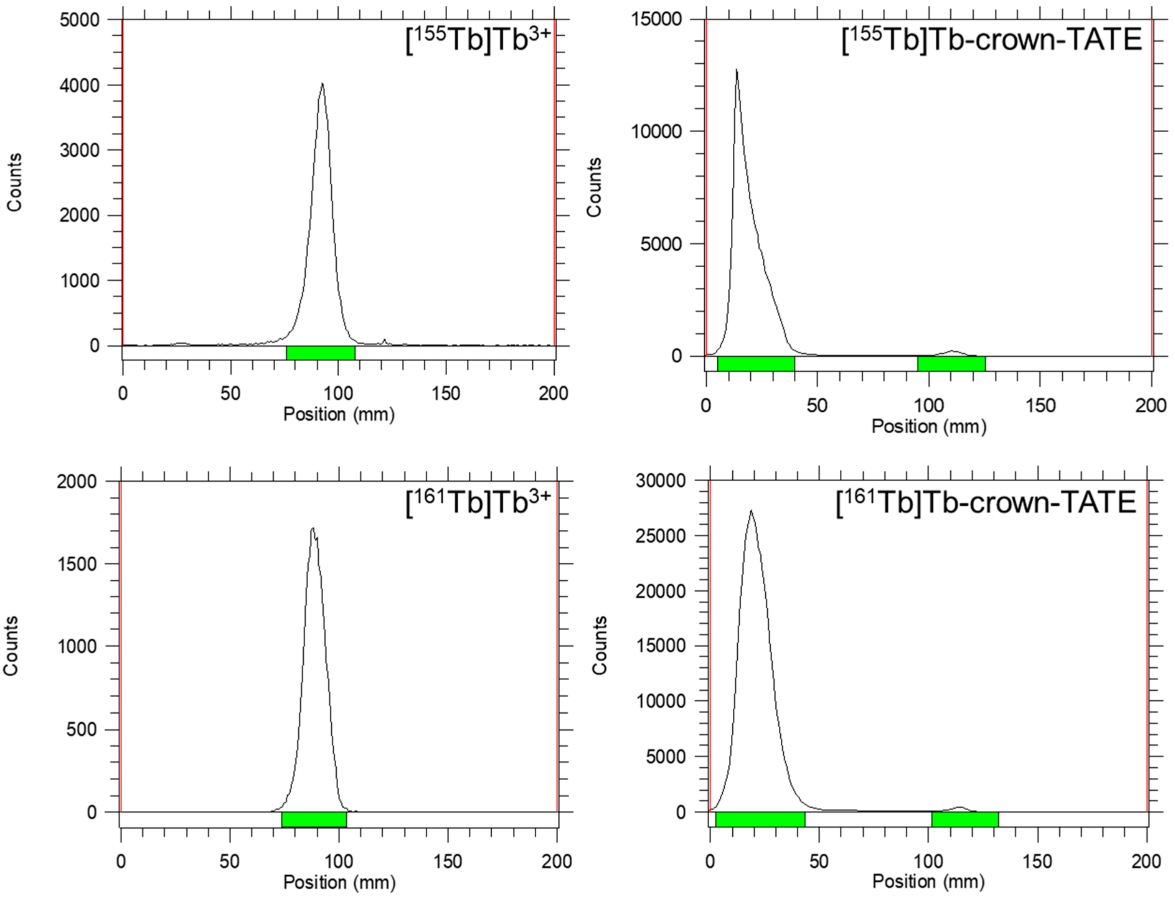

Figure S2. Representative radio-TLC traces for ^{155}Tb and ^{161}Tb – labelled crown-TATE and negative control reactions. Measured using SG-paper iTLC plates with EDTA (50 mM, pH 5.5) as eluent.

Preclinical Studies

SPECT Biodistribution Studies

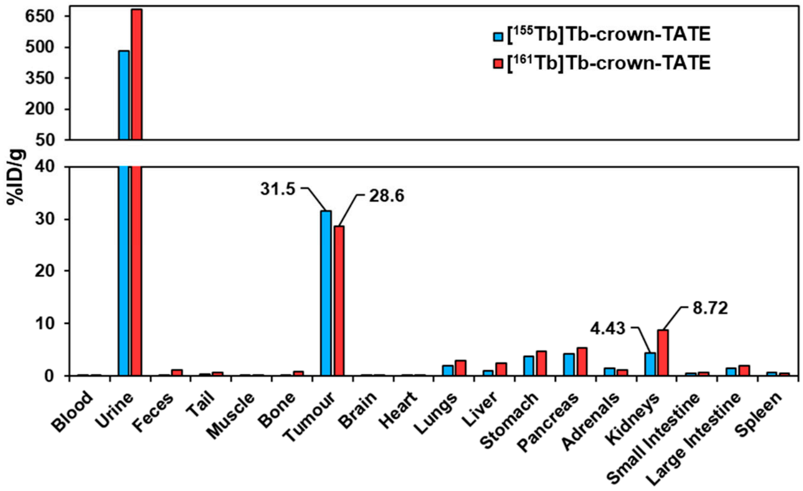

Figure S3. Biodistribution results for imaging mice bearing AR42J tumour xenografts at 2.5 h post-administration of [^{155}Tb]Tb-crown-TATE (13.8 MBq, 0.71 nmol) and [^{161}Tb]Tb-crown-TATE (7.95 MBq, 0.70 nmol).

Table S1. Biodistribution results for imaging mice bearing AR42J tumours xenografts at 2.5 h post administration of [^{155}Tb]Tb-crown-TATE (13.8 MBq, 0.71 nmol) and [^{161}Tb]Tb-crown- TATE (7.95 MBq, 0.70 nmol).

Organ	[^{155}Tb]Tb-crown-TATE %ID/g	[^{161}Tb]Tb-crown-TATE %ID/g
Blood	0.07	0.21
Urine	482.5	683.1
Feces	0.22	1.23
Tail	0.32	0.68
Muscle	0.02	0.04
Bone	0.28	0.83
Tumour	31.51	28.58
Brain	0.02	0.02
Heart	0.10	0.21
Lungs	1.97	2.91
Liver	0.98	2.39
Stomach	3.74	4.78
Pancreas	4.31	5.39
Adrenals	1.42	1.22
Kidneys	4.43	8.72
Small Intestine	0.50	0.75
Large Intestine	1.43	1.99
Spleen	0.70	0.44
Tumour-to-Blood	465	134
Tumour-to-Muscle	1534	650
Tumour-to-Bone	114	34.3
Tumour-to-Kidney	7.12	3.28

Full Biodistribution Studies

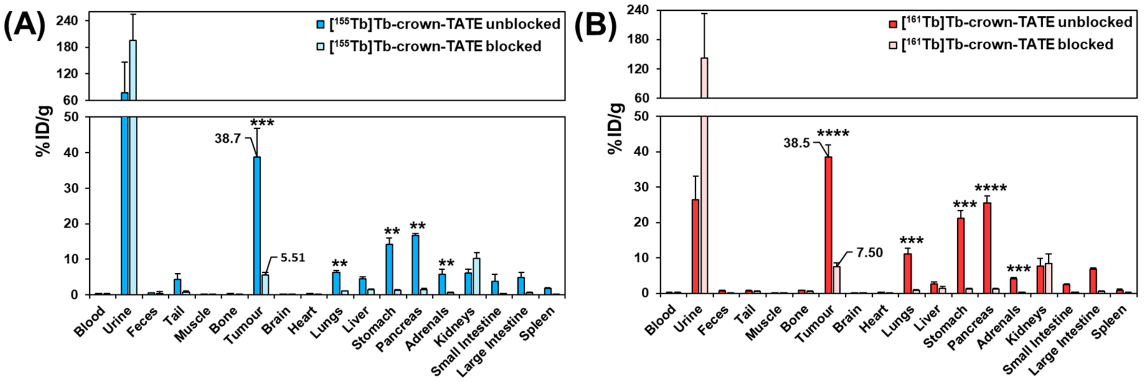

Figure S4. Biodistribution results in male NRG mice bearing AR42J tumour xenografts at 2 h p.i.of: **(A)** [^{155}Tb]Tb-crown-TATE (~175 kBq, 93 pmol/animal) (n=4) (unblocked) and [^{155}Tb]Tb-crown-TATE (~175 kBq, 93 pmol/animal) (blocked with DOTATOC (23 nmol/animal)) (n=3); **(B)** [^{161}Tb]Tb-crown-TATE (~850 kBq, 76 pmol/animal) (unblocked) (n=4) and [^{161}Tb]Tb-crown-TATE (~850 kBq, 76 pmol) (blocked with DOTATOC (23 nmol/animal) (n=3). (** = $p < 0.01$, *** = $p < 0.001$, **** = $p < 0.0001$).

Table S2. Full biodistribution results for [^{155}Tb]Tb-crown-TATE (~175 kBq, 93 pmol/animal) (n=4) (unblocked); [^{155}Tb]Tb-crown-TATE (~175 kBq, 93 pmol/animal) (blocked with DOTATOC (23 nmol/animal)) (n=3); [^{161}Tb]Tb-crown-TATE (~850 kBq, 76 pmol/animal) (unblocked) (n=4); and [^{161}Tb]Tb-crown-TATE (~850 kBq, 76 pmol) (blocked with DOTATOC (23 nmol/animal) (n=3) in male NRG mice bearing AR42J tumour xenografts at 2 h p.i.

Organ	[^{155}Tb]Tb-crown-TATE blocked		[^{155}Tb]Tb-crown-TATE unblocked		[^{161}Tb]Tb-crown-TATE blocked		[^{161}Tb]Tb-crown-TATE unblocked	
	%ID/g	Error	%ID/g	Error	%ID/g	Error	%ID/g	Error
Blood	0.25	0.08	0.28	0.15	0.14	0.07	0.17	0.02
Urine	196	58.0	78.0	68.5	142	91.1	26.5	6.5
Feces	0.41	0.55	0.54	0.02	0.09	0.03	0.63	0.13
Tail	0.72	0.31	4.31	1.54	0.46	0.14	0.70	0.09
Muscle	0.04	0.01	0.08	0.03	0.03	0.01	0.04	0.01
Bone	0.13	0.03	0.34	0.06	0.47	0.10	0.73	0.09
Tumour	5.51	0.73	38.7	8.02	7.50	1.08	38.5	3.45
Brain	0.02	0.01	0.04	0.01	0.01	0.00	0.02	0.00
Heart	0.13	0.02	0.32	0.07	0.12	0.04	0.24	0.02
Lungs	1.13	0.03	6.27	0.59	0.89	0.14	11.1	1.62
Liver	1.40	0.29	4.48	0.49	1.43	0.42	2.63	0.59
Stomach	1.31	0.17	14.2	1.88	1.23	0.18	21.2	2.12
Pancreas	1.50	0.26	19.7	4.30	1.20	0.21	25.5	1.96
Adrenals	0.53	0.18	5.79	1.41	0.21	0.05	4.04	0.44
Kidneys	10.2	1.65	6.13	0.99	8.44	2.68	7.71	2.11
Small Intestine	0.38	0.04	3.85	1.84	0.24	0.09	2.43	0.23
Large Intestine	0.60	0.06	4.84	1.42	0.48	0.08	6.75	0.40
Spleen	0.19	0.06	1.73	0.33	0.16	0.08	0.86	0.29
Tumour-to-Blood	21.8	-	139	-	52.9	-	226	-
Tumour-to-Muscle	136	-	488	-	236	-	1021	-
Tumour-to-Bone	42.2	-	113	-	16.0	-	52.4	-
Tumour-to-Kidney	0.54	-	6.32	-	0.89	-	4.99	-
